# Case Report: A case of severe viral pneumonia complicated by pulmonary embolism treated with extracorporeal membrane oxygenation combined with interventional thrombectomy

**DOI:** 10.3389/fcvm.2025.1680758

**Published:** 2025-12-04

**Authors:** Jiaqi Wang, Bingzhu Hu, Cong Zhang, Yingjie Chen, Shi Chen, Cheng Jiang, Fajiu Li

**Affiliations:** Department of Pulmonary and Critical Care Medicine, The Sixth Hospital of Wuhan, Affiliated Hospital of Jianghan University, Wuhan, Hubei, China

**Keywords:** severe pneumonia, extracorporeal membrane oxygenation, pulmonaryembolism, interventional thrombectomy, case report

## Abstract

We report a case of severe H1N1 influenza pneumonia complicated by intermediate-high risk pulmonary embolism (PE) in a 70-year-old male presenting with dyspnea and fever. Initial chest CT demonstrated bilateral interstitial infiltrates and a throat swab was positive for H1N1 on PCR. Despite aggressive antiviral, antibiotic, and respiratory support, the patient developed refractory hypoxemia with progressively elevated D-dimer levels. Subsequent CT pulmonary angiography confirmed the diagnosis of pulmonary embolism. As a rescue therapy, catheter-directed thrombolysis (CDT) was initiated under veno-arterial extracorporeal membrane oxygenation (ECMO) support. This intervention led to immediate hemodynamic and respiratory improvement, culminating in the patient's full recovery and discharge. This case highlights the critical need to suspect concomitant pulmonary embolism in severe pneumonia and demonstrates the therapeutic potential of ECMO-assisted CDT.

## Introduction

H1N1 influenza is a well-established cause of severe viral pneumonia and acute respiratory distress syndrome (ARDS) (1). Its association with intermediate- to high-risk PE has been less frequently documented in the literature. The significant overlap in clinical manifestations often results in the under-recognition of PE in these patients, potentially delaying life-saving interventions. The coexistence of severe pneumonia and PE carries a substantially elevated mortality risk and presents considerable therapeutic challenges (2). This report analyses the clinical features and therapeutic difficulties in patients with severe pneumonia complicated by PE and assess ECMO-facilitated CDT as a salvage strategy.

## Case presentation

A 70-year-old male was admitted to our hospital with a 10-day history of dyspnea and fever, without accompanying hemoptysis, syncope, chest pain, or abdominal pain. His medical history was notable for coronary artery stent implantation 7 years prior, and he was currently receiving regular pharmacotherapy including aspirin, beta-blocker, and rosuvastatin. The patient had no history of pulmonary disease, hypertension, or diabetes mellitus. On physical examination, his vital signs were as follows: temperature 36.8°C, heart rate 101 beats per minute, respiratory rate 30 breaths per minute, blood pressure 100/55 mmHg, and oxygen saturation 74%–82% under dual-channel oxygen supplementation. Clinical manifestations included cyanosis of the lips, tachypnea, labored breathing and moist rales.

**Figure 1:**
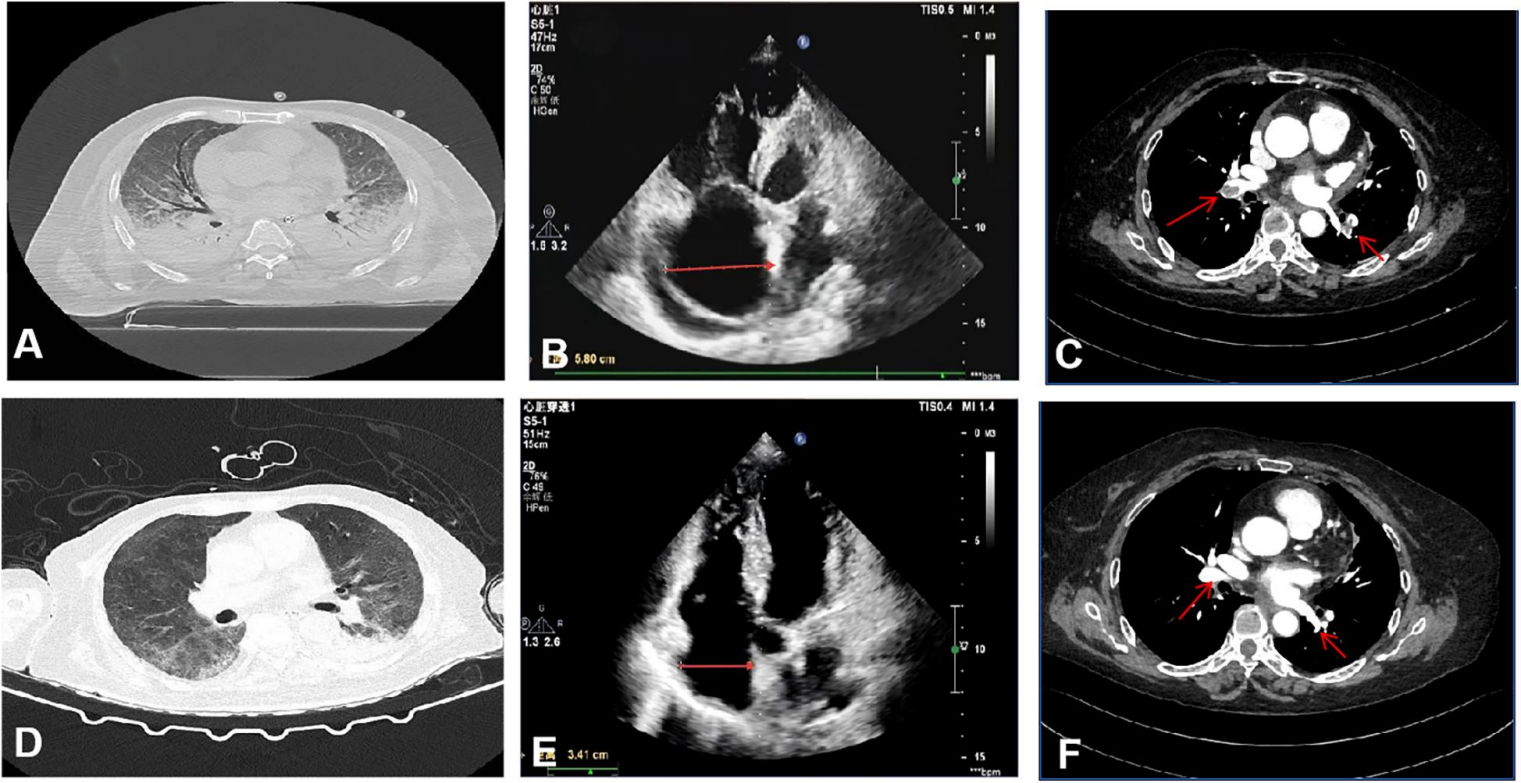
Imaging and echocardiographic findings before and after treatment. **(A–C)** Pre-treatment: **(A)** Chest CT shows bilateral pulmonary consolidation and interstitial infiltrates. **(B)** Transthoracic echocardiography reveals right atrial enlargement (transverse diameter 5.8 cm). **(C)** CT pulmonary angiography demonstrates extensive bilateral pulmonary embolism. **(D–F)** Post-treatment: **(D)** Chest CT indicates resolution of pulmonary consolidation and marked improvement of interstitial opacities. **(E)** Echocardiography shows significant reduction in right atrial size (transverse diameter 3.41 cm). **(F)** CT pulmonary angiography confirms substantial reduction in thrombus burden.

Blood gas analysis showed PH 7.38 (Reference Range 7.35–7.45), PaCO_2_ 30.08 (Reference Range 35–45) mmHg, PaO_2_ 47.84 (Reference Range 80–100) mmHg, lactate 5.66 (Reference Range 1–1.7) mmol/L, and an oxygenation index of 78.4 mmHg (FiO_2_ 0.61). Troponin was 0.125 (Reference Range 0–0.04) ng/mL, NT-ProBNP was 3,735.2 (Reference Range 0–900) pg/mL, and a throat swab PCR test was positive for influenza A H1N1 nucleic acid. Pulmonary CT revealed interstitial infection in both lungs (Figure 1A). Lower extremity ultrasound showed hypoechoic areas within the intramuscular veins of both lower legs, and echocardiography revealed right ventricular enlargement (Figure 1B). Left ventricular systolic function was preserved.

The patient was diagnosed with severe H1N1 viral pneumonia complicated by type I respiratory failure and heart failure, based on integrated clinical, radiological, and laboratory findings. The patient was treated with antibiotics (Empirically administered Cefoperazone-Sulbactam 3 g q8h combined with Moxifloxacin 0.4 g qd), antiviral therapy (Oseltamivir 75 mg bid), anti-inflammatory treatment (Methylprednisolone 40 mg bid), anticoagulation (6000IU QN), non-invasive ventilator-assisted ventilation, and high-flow nasal cannula oxygen therapy (HFNC). On January 15, 2024, the patient's condition deteriorated, with the oxygenation index falling to 48 mmHg, necessitating endotracheal intubation, invasive mechanical ventilation, and prone positioning. Despite these interventions, hypoxemia persisted. Elevated D-dimer levels (6.79 mg/L, Reference Range <0.5) prompted computed tomography pulmonary angiography, which revealed extensive bilateral pulmonary emboli (Figure 1C). Given the critical clinical status, a multidisciplinary team recommended veno-arterial extracorporeal membrane oxygenation (VA-ECMO) combined with percutaneous thrombectomy. On January 16, VA-ECMO was initiated percutaneously via the right femoral vein (21 Fr) and left femoral artery (17 Fr) using a Maquet HLS Set Advanced circuit. Initial settings included a pump speed of 2,500–3,000 rpm, titrated to maintain a blood flow of 2.0–3.0 L/min, and a sweep gas flow of 3.0 L/min with an FiO_2_ of 0.8. A standardized anticoagulation protocol with unfractionated heparin (3,000-unit bolus followed by 750 units/hour infusion) was used, targeting an ACT of 180–200 s. Meticulous catheter site care and daily surveillance for circuit thrombosis or limb ischemia were performed, enabling an uneventful ECMO course. Percutaneous mechanical thrombectomy was performed subsequently. Angiography revealed extensive filling defects involving the LA1, RA8, RA7, RA4, RA5, RA1 and RA2 segmental arteries. Following thrombus aspiration using an Indigo aspiration system, repeat pulmonary arteriography demonstrated restored patency with robust bilateral pulmonary arterial opacification, confirming successful reperfusion. The patient's oxygenation index immediately rose to 90 mmHg postoperatively. On January 23, after the above treatments and supportive care, the patient's oxygenation level significantly improved, reaching 206.5 mmHg. With pulmonary CTA showing a reduction in thrombi and subsequent echocardiography confirming a diminished right atrial size (Figures 1E,F), the patient was successfully weaned from ECMO support. On January 31, Bronchoalveolar lavage fluid cultures later identified multidrug-resistant Acinetobacter baumannii (Multidrug-Resistant), leading to targeted antibiotic therapy with polymyxin 150 mg q12h combined with Tigecycline 50 mg q12h (with an initial loading dose) until discharge. By February 13, follow-up imaging demonstrated significant resolution of pulmonary infiltrates (Figure 1D), and the patient was discharged in stable condition. (See timeline of key interventions and clinical milestones in Table 1, change of biomarker in Table 2).

**Table 1:** Timeline of key clinical events and interventions.

Time	Clinical events and interventions
Jan 13, 2024	Hospital admission & diagnosis of severe H1N1 pneumonia
Jan 15, 2024	Endotracheal intubation due to worsening respiratory failure
Jan 16, 2024	VA-ECMO initiation and subsequent percutaneous pulmonary artery thrombectomy
Jan 23, 2024	Successful weaning from VA-ECMO
Feb 07, 2024	Discharge from the Intensive Care Unit (ICU)
Feb 13, 2024	Discharge from the hospital

**Table 2:** The change of NT-proBNP, cTnI, SaO_2_ (%), HR, D-dimer, platelet count, PT, fibrinogen, APTT and PaO_2_/FiO_2_.

Biomarker/Date	1–13	1–15	1–16	1–23
NT-proBNP (pg/mL)	3,735.2	13,885.8	5,728.6	1,749.8
cTnI (ng/mL)	0.125	0.118	0.933	0.126
SaO_2_ (%)	74	68	96	98
HR (bpm)	101	105	70	66
D-dimer (mg/L)	2.48	6.79	3.99	2.75
PLT (10^9^/L)	147	115	145	139
PT (S)	13.6	16.4	15.5	13.4
Fibrinogen (g/L)	4.23	4.11	3.37	3.45
APTT (S)	34.10	84.10	31.70	40.08
PaO_2_/FiO_2_	78.4	48	90	234.8

NT-proBNP, N-terminal pro-B-type natriuretic peptide; cTnI, cardiac troponin I; PLT, platelet count; PT, prothrombin time; APTT, activated partial thromboplastin time.

NT-proBNP (Reference Range 0–900) pg/mL; cTnI (Reference Range 0–0.04) ng/mL; D-dimer (Reference Range 0–0.5) mg/L; PLT (Reference Range 125–350) 10^9^/L; PT (Reference Range 9.2–13.9) S; Fibrinogen (Reference Range 2–4) g/L; APTT (Reference Range 21.2–34.8) S.

## Discussion

Influenza is an acute respiratory infection with an insidious onset. Early symptoms resemble those of common colds and lung infections, making it highly prone to misdiagnosis as a common cold or pneumonia. The disease progresses rapidly, often developing into severe pneumonia within a short period, leading to respiratory failure, multiple organ failure, and death. The mortality rate for influenza is 5.5%, with the highest rate observed in individuals aged 85 or older at 12.2%. Among all fatal cases, 76.4% involve elderly individuals aged 65 or older (3). Approximately 13%–45% of cases require admission to the intensive care unit (ICU) (4). Influenza can increase the risk of pulmonary embolism.The development of pulmonary embolism in viral infections is multifactorial (5). Viral infection of endothelial cells triggers their activation and injury, initiating a pro-inflammatory response characterized by the release of cytokines and upregulation of von Willebrand factor (VWF) and adhesion molecules. This cascade leads to immunothrombosis, a process amplified by neutrophil extracellular traps (NETs), platelet-derived polyphosphate (PolyP), and complement activation, which synergistically drive the intrinsic coagulation pathway. The outcome is uncontrolled thrombin generation and thrombus formation. Studies indicate that patients with influenza are considerably less likely to develop *in situ* pulmonary thrombosis than those with COVID-19 (6). However, in patients with severe pneumonia, prolonged immobilization can lead to lower extremity deep vein thrombosis (DVT), which may subsequently cause pulmonary embolism (PE). A study showed that the incidence of influenza complicated by pulmonary embolism is 3.3%, which is lower than the 10.95% observed in COVID-19 (7). The incidence of thrombotic events in critically ill influenza ICU patients is significantly higher than in critically ill community-acquired pneumonia (CAP) patients (21.3% vs. 5.7%; *p* < 0.05). Compared to critically ill influenza patients without thrombosis, those with thrombosis exhibited significantly higher rates of mechanical ventilation use, longer duration of mechanical ventilation, longer ICU stay, and increased 90-day mortality (8). The PE in this patient was likely the result of a combination of embolization from a lower extremity DVT and *in situ* thrombosis formation within the pulmonary vasculature.

Extracorporeal membrane oxygenation (ECMO) is an extracorporeal life support technique used to treat severe cardiopulmonary failure. In recent years, the application of ECMO in intensive care and emergency medicine has become increasingly widespread, demonstrating significant efficacy, particularly in treating acute respiratory distress syndrome (ARDS), cardiogenic shock, and high-risk pulmonary embolism (9). According to the latest research and clinical practice, ECMO not only plays a crucial role in supporting vital signs during the acute phase of the disease but also provides necessary stabilization for subsequent surgical interventions (10, 11). A systematic review and meta-analysis, including eight studies and 266 patients treated with ECMO, found that the mortality rate decreased to 28% after ECMO treatment for severe H1N1 infection, suggesting that ECMO is effective for influenza-related ARDS (12–19).

Catheter-directed therapy (CDT) can be used to rapidly reduce the pulmonary arterial thrombus burden (20, 21). According to the 2022 Joint Clinical Consensus Statement of the European Society of Cardiology Working Group on Pulmonary Circulation and Right Ventricular Function and the European Society of Percutaneous Cardiovascular Interventions on the Percutaneous Treatment of Acute Pulmonary Embolism (22), for high-risk pulmonary embolism patients, CDT may be considered if there are contraindications to thrombolysis or if thrombolysis fails (i.e., no hemodynamic improvement 2–4 h after thrombolysis or after completing local thrombolysis). For intermediate-high risk pulmonary embolism patients, CDT may also be considered if no improvement is observed 24–48 h after initial anticoagulation therapy. Current clinical research has also explored the efficacy of CDT in intermediate-risk PE. The ULTIMA randomized controlled trial showed that compared to standard anticoagulation therapy, the EKOS system could more significantly reduce the right ventricular-to-left ventricular diameter ratio in intermediate-risk pulmonary embolism patients within 48 h without increasing the risk of major bleeding. The 2023 FLASH registry study (23), involving 800 intermediate or high-risk pulmonary embolism patients from 50 centers in the United States, found that patients treated with the FlowTriever thrombectomy system experienced rapid clinical improvement, with an in-hospital mortality rate as low as 0.3% and no device-related serious adverse events, indicating good safety. This case demonstrates that for patients with severe ARDS and pulmonary embolism, CDT can be a life-saving intervention by rapidly improving oxygenation and hemodynamics.

Vascular interventional pulmonary arterial thrombectomy under ECMO support is an advanced and highly challenging treatment method. In this procedure, ECMO first provides continuous cardiopulmonary support, ensuring adequate blood supply and oxygenation to vital organs during thrombectomy. Its complex management and potential complications require close collaboration and meticulous management by a multidisciplinary team (24). Early identification and management of complications are crucial for improving patient outcomes (25). In a single-center study of 15 patients with high-risk pulmonary embolism who received combined CDT and ECMO, ECMO was successfully discontinued after a mean of 5.4 days, with only one mortality.This study provides preliminary evidence for the feasibility of percutaneous large-bore aspiration embolectomy in combination with VA-ECMO support in patients with high-risk PE (26). Our case highlights the potential benefit of early ECMO-supported CDT for PE in patients with ARDS, indicating a promising direction for future management.

## Conclusion

ARDS with concomitant PE, particularly of viral etiology, may represent a fundamentally different pathophysiological entity requiring a combined cardiopulmonary support strategy. Future comparative studies of ECMO with vs. without CDT in ARDS/PE overlap syndrome are imperative to validate this approach and refine patient selection criteria for this aggressive intervention.

## Data Availability

The raw data supporting the conclusions of this article will be made available by the authors, without undue reservation.
